# Towards a machine-readable literature: finding relevant papers based on an uploaded powder diffraction pattern

**DOI:** 10.1107/S2053273322007483

**Published:** 2022-08-19

**Authors:** Berrak Özer, Martin A. Karlsen, Zachary Thatcher, Ling Lan, Brian McMahon, Peter R. Strickland, Simon P. Westrip, Koh S. Sang, David G. Billing, Dorthe B. Ravnsbæk, Simon J. L. Billinge

**Affiliations:** a Department of Applied Physics and Applied Mathematics, Columbia University, New York, NY, 10027, USA; b Department of Physics, Chemistry and Pharmacy, University of Southern Denmark, DK-5230 Odense M, Denmark; c International Union of Crystallography, Chester, CH1 2HU, UK; d School of Chemistry, University of the Witwatersrand, Private Bag 3, PO WITS, 2050, South Africa; e Department of Chemistry, Aarhus University, DK-8000 Aarhus C, Denmark; f Condensed Matter Physics and Materials Science Department, Brookhaven National Laboratory, Upton, NY, 11973, USA; Institute of Crystallography - CNR, Bari, Italy

**Keywords:** machine-readable scientific literature, data-driven literature search, powder diffraction, data similarity, CIF

## Abstract

A prototype application for machine-readable literature is investigated. The program is called *pyDataRecognition* and serves as an example of a data-driven literature search, where the literature search query is an experimental data set provided by the user. The user uploads a powder pattern together with the radiation wavelength. The program compares the user data to a database of existing powder patterns associated with published papers and produces a rank ordered according to their similarity score. The program returns the digital object identifier and full reference of top-ranked papers together with a stack plot of the user data alongside the top-five database entries. The paper describes the approach and explores successes and challenges.

## Abstract 2

A prototype application, *pyDataRecognition*, is described and tested. It has the goal that, given a measured powder diffraction pattern, it will return a list of publications from the IUCr Journals database that might be related based on the similarity to powder diffraction data deposited for those publications. This explores the possibility of a machine-readable literature where, for example, relevant studies may be found automatically through data similarity matches of online databases.

## 1. Introduction

The activity of communicating science, including paper writing, always includes a search of the literature to discover and acknowledge prior work (Garfield, 1996 ▸). Since the advent of the internet, this process has largely moved from manual, library-based searches to online searches using search engines (Butler, 2000 ▸). Literature search engines such as Google Scholar (Van Noorden, 2014 ▸) normally work by accepting text and metadata search queries, such as author names, keywords, journal name, year, and so on. In contrast, here we explore the concept of a data-seeded literature search where we use a measured data set as the search query to retrieve data-relevant papers from the literature. We chose to use X-ray powder diffraction data for our test case.

X-ray powder diffraction is an important technique in materials science, where structural characterization is at the very centre of the workflow as it is inherently linked to material properties. The goal of the technique is to understand the arrangement of atoms in the material based on measurements of X-ray (or neutron or electron) diffraction. When the sample is a powder, the resulting diffractogram is a 1D pattern of peaks called a powder diffraction pattern (Gilmore *et al.*, 2019 ▸; Dinnebier & Billinge, 2008 ▸). This serves as a 1D fingerprint of the structure of the material.

The challenge for our purposes is that there are no large open databases of experimental powder data. The crystallography community recognized early the need for structured data related to chemical structure and developed the crystallographic information framework (CIF) (Hall *et al.*, 1991 ▸). This was extended to allow for the capture of experimental data from powder experiments as part of the powder CIF development (Hall *et al.*, 2006 ▸). CIF dictionaries provide machine-readable definitions of data items that can appear in a CIF-structured database such as a CIF file. Such CIF files (or ‘CIFs’) form the basis for multiple chemical [ICDD (International Centre for Diffraction Data) (Gates-Rector & Blanton, 2019 ▸), ICSD (Inorganic Crystal Structure Database) (Zagorac *et al.*, 2019 ▸), CSD (Cambridge Structural Database) (Groom *et al.*, 2016 ▸), COD (Crystallography Open Database) (Gražulis *et al.*, 2009 ▸)], macromolecular [wwPDB (Worldwide Protein Data Bank) (Berman *et al.*, 2000 ▸)] and materials science [Materials Project (Jain *et al.*, 2013 ▸)] structural databases.

However, of submitted CIFs that contain data resulting from a powder diffraction study, few include the associated diffractogram data (indeed, one desired outcome of this work would be to increase the incentives for authors to include the underlying diffractogram data). The journals of the International Union of Crystallography (IUCr) archive all CIFs uploaded by authors with subsequently published papers. From this database we were able to extract a relatively small subset of CIFs that do contain powder patterns, along with metadata that allow the related paper to be found. This subset (787 CIFs) is the database we use for testing our prototype.

As a simple illustration of the kind of benefits that may derive from having a machine-readable capability for the contents of literature papers, we develop here a prototype application that would help experimentalists carry out a literature search early in the process of their study. The specific use case that we want to demonstrate is described below, but the vision is a software application that takes a measured powder pattern as input and returns a list of relevant papers in the existing literature, preferably without the user having to upload a significant amount of, or ideally any, additional information about the experiment. This is reminiscent of modern facial recognition capabilities but it is experimental data recognition and so we call the Python language based prototype application *pyDataRecognition*.

## 2. Machine-readable versus human-readable literature

For the past ∼350 years, the main goal of the scientific literature has been to condense scientific understanding into documents that are intelligible to humans. It has been enormously successful by any measure. However, the literature is growing rapidly and it is becoming difficult for humans to keep up with the number of new publications. Also, it becomes hard to assimilate so much information and find correlations and insights between related studies. When given structured data, machines are very good at finding correlations and clustering by similarity as exemplified by facial recognition algorithms (Anwarul & Dahiya, 2020 ▸). There is a type of machine learning whereby machines read papers that were written for humans to understand, a process called natural language processing (NLP) (Chowdhary, 2020 ▸). However, this process, whilst valuable for extracting information from the historic canon, is not the best way for machines to assimilate information from data. We can expect much greater efficiencies in machine processing of the scientific information if we can take steps to make scientific papers readable by machines directly.

For this process to succeed, we need data in papers to be in accessible and structured data formats and saved with sufficient metadata to give important contextual information. The human being has a very highly developed capability for pattern recognition. When we write a human-readable paper, we take our data and make an image, for example, by plotting the result as a line plot. It is much easier for the human reader to see similarities and derive insights from the data plotted as an image, but this is hard for the machine. A literature that is written to be read by both humans and by machines would also have the data that were used to form the image saved in a machine-readable way, with important metadata such as what is being plotted, the quantity and units of both the *x* and *y* arrays, the sample that was measured to produce the plot, the people who did the work, and so on. This is rarely done currently but is needed to realize the benefits of machine-learned science.

## 3. Prototype literature search application

In order to explore the kind of benefits that might be derived by having a machine-readable literature, we explore a very simple use case that makes use of a (small) database of structured, tagged, experimental data and does something useful with it. The simple use case we explore is that of a measured data set used as the input in a literature search.

### 3.1. Use case

The use case is described in the following way. A structure scientist has a powder diffraction pattern from a particular sample collected on their powder diffractometer. They upload the data to the search application, together with a limited amount of relevant experimental information. The application then will search a database of stored powder diffraction data associated with published papers. It will then return a list of relevant papers based on the similarity between the data uploaded by the user and the powder patterns appearing in the papers. In the simple first iteration of the concept, the relevance will just be a ranking based on the similarity between the powder patterns in these papers and the powder diffraction data uploaded by the user.

The advantage of this use case is that the IUCr has a database of experimental powder patterns in a machine-readable powder-CIF format (Hall *et al.*, 2006 ▸) that have been deposited by authors at the same time as they submitted the paper to the relevant IUCr journal. These are the experimental data that generally appeared as images in figures in the linked papers. The existence of this structured database of experimental powder patterns linked to published papers is therefore a valuable resource for prototyping the approach.

We note that there are several databases that facilitate computational literature searches including CrossRef (Crossref, 2020 ▸), Scopus (Mongeon & Paul-Hus, 2016 ▸; Burnham, 2006 ▸), Web of Science (Mongeon & Paul-Hus, 2016 ▸; Mikki, 2009 ▸), arXiv (Ginsparg, 2011 ▸), Google Scholar (Mikki, 2009 ▸; Samadzadeh *et al.*, 2013 ▸), Google Image Search (Fergus *et al.*, 2005 ▸), and so on. The purpose of this work is to show how properly tagged data held in a structured database can be included in literature search workflows, helping scientists to do better science more quickly.

### 3.2. Software implementation

The use case presented above has been implemented in a Python package. The package uses home-written functions based on well established third-party libraries like *NumPy* (Harris *et al.*, 2020 ▸), *Matplotlib* (Hunter, 2007 ▸), *SciPy* (Virtanen *et al.*, 2020 ▸), *scikit-beam* (scikit beam, 2022 ▸) to complete the use case.

To run the program, the user must provide the diffraction data for which the query should run. Currently, the data should be provided as a two-column text file, possibly with a header, of intensity versus an independent variable. The independent variable may be in the form of diffraction angle, 2θ in °, *d* spacing, in Å, or the momentum transfer, *Q*, in Å^−1^. If the independent variable is 2θ, the X-ray or neutron wavelength in Å also needs to be provided. All comparisons between data within the program are done with a *Q* independent variable. It will be straightforward to support different file formats in a production version of the code later.

The program then uses a distance metric to determine the similarity of the uploaded pattern to every pattern in the database. In the current implementation we are using the Pearson correlation (Pearson & Galton, 1895 ▸), *r*_*xy*_, 

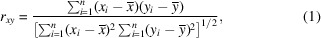

where *x* and *y* are 1D arrays of equal size, and 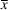 and 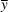 are their means, respectively. The value of the correlation coefficient can vary between +1 and −1. A value of +1 means the two data sets are identical (perfect positive correlation), 0 implies no correlation between the data sets. Numbers less than zero imply negative correlation. It is calculated using the pearsonr() method within the *scipy.stats* package (Virtanen *et al.*, 2020 ▸). Since our goal is to find similar data, we seek diffraction patterns with *r*_*xy*_ close to 1.

For a comparison of two data sets using the Pearson correlation, the two intensity arrays need to be on the same *Q* grid. In general, powder patterns are measured over different ranges of *Q* and on different arbitrary *Q* grids. To address this issue, we automatically determine the *Q*-space overlap region of the user and database data sets and linearly interpolate the data onto a common regular *Q* grid in this interval. Currently, a step size of Δ*Q* = 10^−3^ Å^−1^ is used. The user-supplied and target intensity arrays are then linearly interpolated onto this grid and the Pearson correlation is computed. Currently, the comparison is done over the full overlapping range as long as there is at least a 20 nm^−1^ overlap. If the overlap is smaller than this the database entry is not considered. As a result of this heuristic, similarities are compared between pairs of data computed over different ranges of overlap. The Pearson measure is normalized by the number of points that are computed, making comparisons between overlap regions of different length possible. This seems to give reasonable results but could be revisited in the future.

The process of finding the overlapping range in *Q* space, calculating a regular *Q* grid, doing linear interpolation, and conducting Pearson correlation analysis between the user data and the data from a CIF is done for every CIF in the *pyDataRecognition* database. This is possible because of the small size of the database but will not scale to large databases of data and more efficient approaches will be investigated in the future.

The program then determines a rank-ordered list based on similarity, and extracts from the database entry metadata the digital object identifier (DOI) (Paskin, 1999 ▸) of the paper that is associated with the the ranked data set. The full reference of the associated paper is determined by making an API call to CrossRef (Crossref, 2020 ▸) using the DOI. The rank-ordered list is then returned to the user containing the rank, the Pearson *r* value, the DOI and the full paper reference. This information is also saved to a text file.

**Figure 1:**
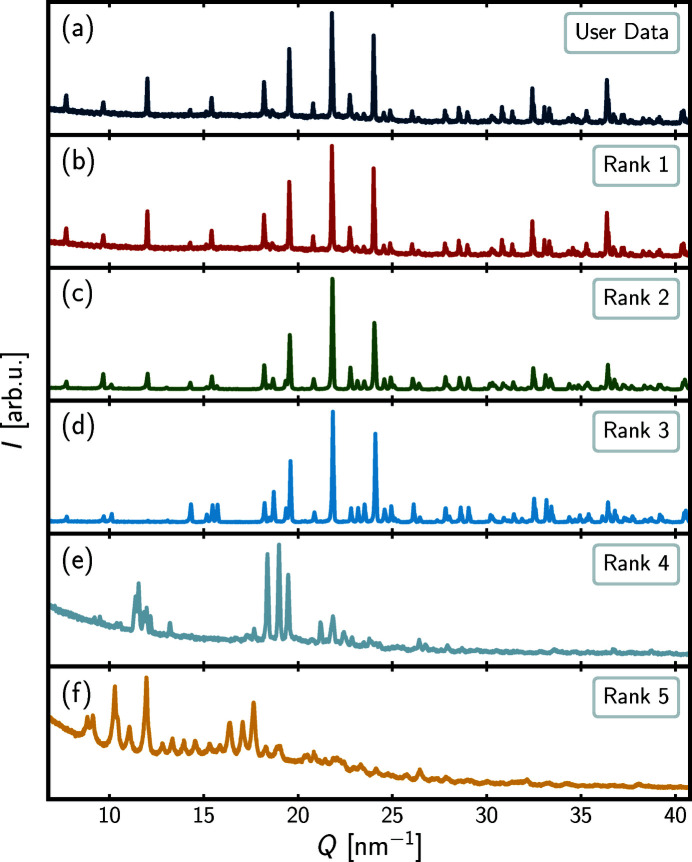
Intensity, *I*, in arbitrary units as a function of momentum transfer, *Q*, in nm^−1^, for the user data (topmost) and the top-five *pyDataRecognition* database entries in descending order. The full range of the user data is shown, whereas only the comparison region is shown for each database entry. The rank scores, DOIs and references can be found in Table 1 ▸.

**Figure 2:**
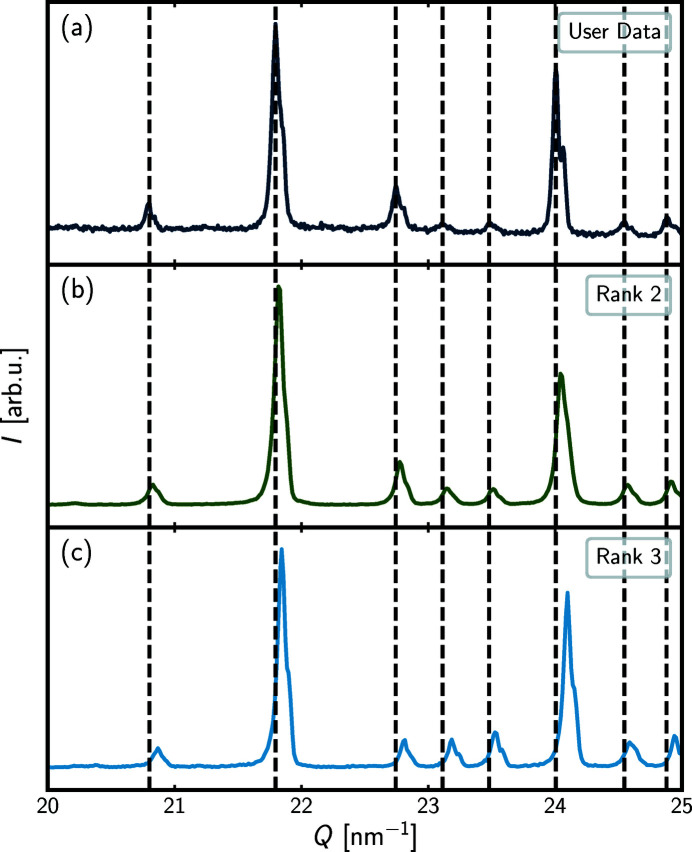
Intensity, *I*, in arbitrary units as a function of momentum transfer, *Q*, in nm^−1^, for the user data (topmost) and the rank-2 and -3 *pyDataRecognition* database entries. The data are plotted for the *Q* range from 20 to 25 nm^−1^. The vertical lines indicate the Bragg positions of the user data.

**Figure 3:**
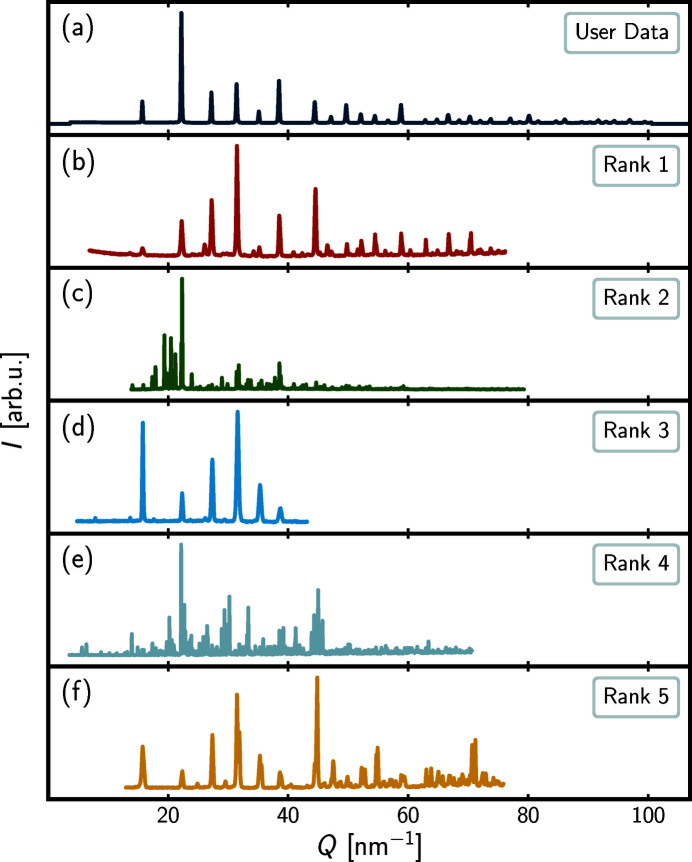
Intensity, *I*, in arbitrary units, arb.u., as a function of momentum transfer, *Q*, in nm^−1^, for the user data (topmost) and the top-five *pyDataRecognition* database entries in descending order. The full range of the user data is shown, whereas only the region used for comparison is shown for each database entry. The rank scores, DOIs and references can be found in Table 2 ▸.

The five most similar *pyDataRecognition* database entries are plotted together with the user data to enable the user to visually inspect similarities between the data sets. Examples of output rank-ordered lists are given in Tables 1–3 and plots in Figs. 1–3 in Section 4.1.

## 4. Outcomes

### 4.1. Results

At the moment of writing, ∼515 valid CIFs, out of 785 total in the *pyDataRecognition* database, are included in the analysis. They originate from ∼215 papers. The actual number of CIFs and papers included in the analysis depends on the *Q* range of the user data, as a minimum shared *Q* range of 20 nm^−1^ between the user and database patterns is required for the CIF to be included in the analysis.

Here, we explore the performance of the prototype *pyData­Recognition* with a number of query examples. The first example serves to test that the algorithm finds, with top rank, a data set that actually exists in the database. The second example is a better test of the real use case. We provide real data but choose a very common structural form (perovskite) with the expectation that there will be representatives of this structure from more than one sample and composition, even given the limited size of the current database of 785 CIFs. In the third example, we provide a neutron data set as user data to explore how the program behaves when provided neutron data as input whilst the data in the database come predominantly from X-ray data.

*Query example 1*. For the first example, the user data are taken from a CIF from the paper by Stähli *et al.* (2016 ▸). The paper is on hydrogen-substituted β-tricalcium phosphate synthesized in organic media, *i.e.* a Mg-free whitlockite, represented by the formula Ca_21−*x*_(HPO_4_)_2*x*_(PO_4_)_14−2*x*_, where *x* = 0.80 ± 0.04. The data are from an X-ray experiment. As the user data are taken from a database entry, the expected outcome of the query is to have a perfect match, *i.e.* a score of 1, *r*_*xy*_ = 1. From Table 1 ▸, it can be seen that the test went well, and a perfect match is found for a CIF appearing in the paper by Stähli *et al.* (2016 ▸).

In Figs. 1 ▸(*a*) and 1 ▸(*b*), visual inspection confirms that the plots of the user data and the rank-1 database entry are identical.

It is encouraging that the program returns the paper from which the user data were derived as the top-ranked result.

Moving down in Table 1 ▸, the rank-2 entry (Zatovsky *et al.*, 2010 ▸), which studies Rietveld refinement of whitlockite-related K_0.8_Ca_9.8_Fe_0.2_(PO_4_)_7_, scores 0.7379. The score indicates an intermediate level of similarity to the user data. Visual inspection of the plot in Fig. 1 ▸(*c*) confirms the similarity and the structural relation between the user data and the rank-2 entry that are both whitlockite-related. Multiple Bragg positions are shared between the two data sets, as reflected in the intermediate score, but at the same time dissimilarities are also present, such as differences in relative intensities and peak splitting, *e.g.* right above 10 nm^−1^, as should be expected from different chemical compositions. The data-driven nature of the *pyDataRecognition* query enables the user to discover other papers with possible relevance to their uploaded data.

The rank-3 data set has a much lower agreement factor (0.4631), than the rank-2 one (0.7379) which might suggest that it is structurally unrelated. However, visual comparison of the diffraction curves [Figs. 1 ▸(*c*) and 1 ▸(*d*)] suggests that there are many similarities between these data sets. In fact, the rank-3 data set (Strutynska *et al.*, 2013 ▸) is from a Rietveld refinement study of a sample isostructural to the mineral whitlockite, AgCa_10_(PO_4_)_7_, which is closely related to the user data set and would certainly be of interest to the user. In this case the Pearson measure seems not ideal as a similarity metric for the current use case.

To explore the origin of the large drop in similarity score between the isostructural rank-2 and -3 samples, in Fig. 2 ▸ we have plotted the user data together with the rank-2 and -3 database entries on an expanded *Q* scale from 20 to 25 nm^−1^ with vertical lines indicating the peak positions of the user data.

We see that there is a small offset in peak position for the rank-2 database entry relative to that of the user data, whereas the offset is more pronounced for the rank-3 database entry. This *Q* offset is likely to explain the low Pearson score of the rank-3 entry. The difference in scores for the rank-2 and -3 database entries gives a hint at how sensitive the current Pearson similarity metric is towards an offset, whether it is an experimental artefact or has a structural origin such as different lattice parameters of otherwise similar structures. This is undesirable behaviour in our similarity metric that we explore further below.

The rank-4 and -5 entries in Table 1 ▸ and Figs. 1 ▸(*e*)–1 ▸(*f*) in the current example appear visually very dissimilar to the user data and are unlikely to be of any interest to the user. However, it is observed that the Pearson scores are quite similar to that of the rank-3 entry that is isostructural. This is further evidence of a weakness in the use of the Pearson metric in the current application as it cannot distinguish an isostructural but shifted pattern from a completely dissimilar pattern. The code was designed for it to be easy to implement different similarity metrics in principle, and finding the best similarity metrics will be an ongoing process.

*Query example 2*. For the second example, the input data are synchrotron X-ray data of the perovskite BaTiO_3_. Since the structural family of perovskites is common, it is hoped that even the current small database will return one or more papers with data from perovskite or perovskite-related structures. The results from the query are found in Table 2 ▸ and Fig. 3 ▸.

From the scores reported in Table 2 ▸, it is evident that no highly similar database entries are encountered as all scores *r*_*xy*_ < 0.6. However, a visual inspection of the top-ranked powder pattern in Fig. 3 ▸(*b*) does show some similarity in peak frequency and positions, so the rank-1 entry may be related to the user data despite the modest score of 0.5723 reported in Table 2 ▸. Looking into the paper (Iturbe-Zabalo *et al.*, 2013 ▸), the topic is symmetry-mode analysis of the phase transitions in SrLaZnRuO_6_ and SrLaMgRuO_6_ ordered double perovskites, *i.e.* a paper on perovskite-derived structures, which is encouraging, considering that the user data were for the perovskite BaTiO_3_; thus, from all of the 514 entries in the database, *pyDataRecognition* has returned a paper describing related data in the top-rank position, albeit with a low similarity score.

Returning to the remaining results reported in Table 2 ▸, it is seen that all scores are <0.4, indicating low Pearson similarity to the user data. For the rank-2 and -4 entries, the low scores seem to reflect structural dissimilarity as the diffraction patterns are visually very different. The rank-2 entry (Sciau *et al.*, 1999 ▸) considers the structures of the paraelectric and ferroelectric phases of Pb_2_KNb_5_O_15_ with orthorhombic symmetry which does appear to be perovskite-related. Fig. 3 ▸(*c*) shows that the database entry possesses a much larger peak density compared with the user data, as also reflected in the rather low score of 0.3906.

The paper of the rank-4 entry (Kasunič *et al.*, 2011 ▸) is on the structure of LaTi_2_Al_9_O_19_, a non-perovskite compound isostructural to SrTi_3_Al_8_O_19_, and so the low score of 0.2881 again reflects a structural dissimilarity. However, the low Pearson scores for the rank-3 and rank-5 results seem surprising, as in these cases the data have a visual resemblance to the user data in Figs. 3 ▸(*a*), 3 ▸(*d*) and 3 ▸(*f*), especially in the rank-3 case. The paper of the rank-3 entry (Orayech *et al.*, 2015 ▸) considers mode-crystallography analysis of the crystal structures and the low- and high-temperature phase transitions in the Na_0.5_K_0.5_NbO_3_ cubic perovskite. This paper clearly describes a closely related structure and we would hope that the *pyDataRecognition* algorithm would find it with a high ranking yet it does not. In the case of the rank-5 entry (Zhang *et al.*, 2009 ▸) it also describes perovskite structures (K_0.05_Na_0.95_NbO_3_ and K_0.30_Na_0.70_NbO_3_).

As was the case for the first query example, the low scores of the otherwise visually similar rank-3 and -5 entries may be explained by a small offset in the lattice parameters. For both entries, the offset is towards higher *Q* values, compared with the user data, which is the likely cause of the poor Pearson score. In addition, there are also clear differences in relative peak intensities compared with the user data and some peak broadening, *e.g.* at 32 nm^−1^, which will also affect the Pearson score.

The rank-3 and -5 entries represent additional false negative results. These results did show up in the top-five list despite their low Pearson scores, which is encouraging, but it is likely that other related papers are being missed with low Pearson scores because of the poor performance of Pearson for the job in hand.

**Figure 4:**
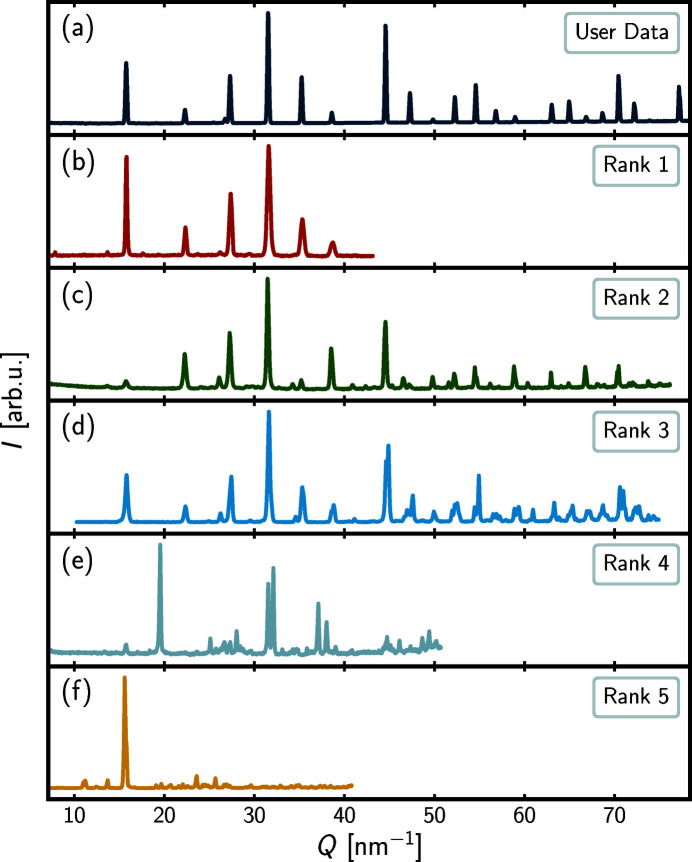
Intensity, *I*, in arbitrary units, arb.u., as a function of momentum transfer, *Q*, in nm^−1^, for the user data (topmost) and the top-five *pyDataRecognition* database entries in descending order. The full range of the user data is shown, whereas only the region used for comparison is shown for each database entry. The rank scores, DOIs and references can be found in Table 3 ▸.

*Query example 3*. The third and last query example reported here is regarding user data for which neutrons were used as the probe, in contrast to the two former query examples that originated from X-ray probes. *pyDataRecognition* accepts powder patterns from any source, X-ray, neutron or electrons, and currently the user is not asked to provide the type of probe on input, just as the type of probe is not regarded when running the query. Regardless, in principle, we would still like the program to return papers describing similar structures. The results are shown in Table 3 ▸ and Fig. 4 ▸.

The user data set is measured for a cubic perovskite sample, (K_0.48_Na_0.48_Li_0.04_)Nb_0.98_Mn_0.02_O_3_ (Mgbemere *et al.*, 2017 ▸), like the second query example above. From Table 3 ▸, a relatively high score of 0.8808 is obtained for the rank-1 database entry (Orayech *et al.*, 2015 ▸). The paper for this entry is on Na_0.5_K_0.5_NbO_3_ perovskite and reports neutron data, explaining the high degree of similarity. The rank-2 (Iturbe-Zabalo *et al.*, 2013 ▸) and -3 (Zhang *et al.*, 2009 ▸) database entries have slightly lower Pearson coefficients (0.7785 and 0.6855, respectively) and the visual similarity between the two is evident, though the rank-3 data set is observed to have lower visual similarity to the user data set. Mostly peaks are in the same place but relative intensities are quite different and peaks are split. The rank-2 database entry was for neutron diffraction data of the double perovskites, SrLaZnRuO_6_ and SrLaMgRuO_6_, encountered before, and indeed weak additional superlattice peaks from the ordering are evident in the pattern. The rank-3 database entry describes neutron data precisely from perovskite K/Na niobate materials, like the user data, albeit with different K:Na ratios, K_0.05_Na_0.95_NbO_3_ and K_0.30_Na_0.70_NbO_3_, as well as the absence of Mn. In this case, peak splitting indicates a symmetry lowering in the database data, but it was correctly detected as closely related by the Pearson similarity in this case. The rank-3 paper (Zhang *et al.*, 2009 ▸) was also encountered as the rank-5 entry in Table 2 ▸ of the second query example. However, the data plotted in Fig. 3 ▸(*f*) stem from a CIF considering K_0.30_Na_0.70_NbO_3_ at 200 K, whereas the data plotted in Fig. 4 ▸(*d*) stem from a CIF considering the material at 180 K and the temperature difference may explain the slight visual differences when comparing the two database entries with one another.

Finally, the very low scores of the rank-4 and -5 entries in Table 3 ▸ are reflected by the observed dissimilarity to the user data in Figs. 4 ▸(*e*) and 4 ▸(*f*) and in this case are due to a structural dissimilarity. The rank-4 entry (Bereciartua *et al.*, 2012 ▸) describes the system Bi_2(*n*+2)_Mo_*n*_O_6(*n*+1)_ (*n* = 3, 4, 5, 6) and the rank-5 entry (Palacios *et al.*, 2003 ▸) [(CH_3_)_4_N](ClO_4_) at low temperature, neither of which are perovskite-related structures.

Overall, the approach is working, with *pyDataRecognition* successfully suggesting to the user, from a database of 785 data sets (of which ∼600 are usable), the same three perovskite structures in comparison with user inputs from perovskite structures, working for both X-ray data from BaTiO_3_ and neutron data from a perovskite Na/K niobate. However, the test revealed certain difficulties that the Pearson correlation coefficient was having at correctly identifying and ranking nearby structures, especially when there was a small shift in the peaks due to different lattice parameters.

### 4.2. Challenges and opportunities

The completed use case demonstrates a proof of concept and reveals the great potential of a machine-readable literature. There is still some way to go before it becomes a practical tool but the prototype highlights some of the challenges as well as the opportunities.

Currently the biggest limitation we encounter is the small database size. Of all the CIFs in the IUCr database (currently numbering around 100 000) only ∼1000 contained experimental powder patterns. In part this is because many studies did not involve the use of powder diffraction data, but also there is limited adoption by authors of the ability to store the actual powder data. Although, through the CIF mechanism, the IUCr is a leader in capturing the powder diffraction data of authors in a structured way, the uptake by the community is still limited. This is currently a focus of the Commission on Powder Diffraction of the IUCr, where new tools for validating deposited CIFs for the contained powder data, and tools for visualizing deposited data are being developed. This *pyDataRecognition* prototype application gives additional incentive to authors as it will clearly make their work more discoverable in the future, and it is only a first step of what can be done if structured data are stored along with the papers describing them.

The current similarity metric, the Pearson correlation coefficient, is a good first step as a similarity measure that can easily be implemented in this prototype application. However, Section 4 illustrates that the job currently done by the Pearson correlation does not completely meet the requirements of the *pyDataRecognition* program, as it is observed that the Pearson correlation seems quite sensitive to *Q* offset. This results in rather low ranking of otherwise visually similar patterns, which is undesirable from a user’s point of view. For experimental data, experimental artefacts from the instrument and the sample are expected, including *e.g. Q* offset. For *pyDataRecognition*, it is desirable to have a similarity metric that tolerates the presence of experimental artefacts and still ranks otherwise similar patterns high to each other. Apart from *Q* offset, potential experimental artefacts to be tolerated include *e.g.* peak broadening from the instrument as well as sample, small peak splittings indicating slight losses in symmetry, and, to some degree, variations in relative intensities of peaks as expected when comparing neutron with X-ray data, or from isostructural but compositionally different samples that may still be relevant to the user. A better similarity metric for the current job required by *pyDataRecognition* would tolerate these aberrations and ideally return results by relevance, much like a Google search does, given the right search query. We will explore different metrics, including ones specifically proposed for powder data (de Gelder *et al.*, 2001 ▸), but this is a big area of research (vom Brocke *et al.*, 2015 ▸) and extending the metric is beyond the scope of the current article.

The prototype also highlights another challenge, which is maintaining the quality of the deposited data and the attached metadata. Despite the small database size, a significant number of the deposited CIFs containing measured data were unusable. Out of 787 CIFs, 785 could be parsed using the CIF parser CifFile.ReadCif() from the *pyCIFRW* Python module (Hester, 2006 ▸). Of those parsed, multiple CIF keys had to be browsed for 2θ, intensity and wavelength values, the main reason being that CIF handles both measured, processed and calculated data. The pdCIF dictionary makes it easier for developers to find the right keys, at least if the use of the keys follows the pdCIF guidelines (Toby, 2006 ▸). However, from the current work, this does not seem to be the case in many instances, the result being that more CIF keys have to be browsed than if the CIF contributors strictly followed the pdCIF guidelines from the IUCr. It may be beneficial to demand that CIF contributors obey the pdCIF guidelines, as this will reduce the number of keys to be browsed in the light of any machine-readable literature effort.

For 59 of the 785 CIFs, the wavelength was missing, preventing a conversion to a physics-based independent variable such as *Q*. Furthermore, for 164 CIFs, the 2θ values were either not stated explicitly or could not be calculated using a CIF-supplied minimum, maximum and step size in 2θ. Whilst it might be possible to modify the algorithm to guess at a resolution for the inconsistent min-max-step calculation, for example, by ignoring the author-supplied bin size and computing it from the minimum and maximum 2θ values and the number of entries in the intensity array, this is not preferred behaviour as it is modifying the user data in possibly ambiguous ways, and so in these cases the CIFs were discarded. Inconsistent size of the min-max-step calculated 2θ array relative to that of the intensity array was encountered for 92 out of all the 164 CIFs. A min-max-step calculated 2θ array consistent with the size of the intensity array was obtained for 120 out of the 785 CIFs processed. None of the CIFs in the current database had *x*-axis data stored in *Q* or *d* quantity, though this would be supported if encountered.

These challenges highlight the need for better validation of CIF inputs of experimental data, as well as more intuitive and easy-to-use tools for experimenters to upload their experimental data and provide the needed metadata.

### 4.3. Software

The open-source Python code for the prototype application *pyDataRecognition* is available through the following public GitHub repository: https://github.com/Billingegroup/pydatarecognition (Özer *et al.*, 2022 ▸).

### 4.4. Next steps

The work described here is an early prototype for a data-driven literature search to illustrate the potential for the machine-readable literature concept. It has served to illustrate the concept and to explore what some of the challenges will be to bring this to fruition. An obvious next step would be to increase the size of the database. We are working with members of the Commission on Powder Diffraction at the IUCr to find ways to increase the amount and readability of powder diffraction data being deposited with submitted papers. In the meantime, one approach to increasing the size of the database is to simulate powder patterns from all structures (including those solved from single-crystal data) in the IUCr CIF database. Comparisons could then be made between uploaded data and simulated, as well as experimental, patterns.

Another issue is finding similarity between diffraction patterns that were measured from the same material but measured under different experimental conditions of instrument resolution and so on. It will be interesting to explore using different deconvolution methods and different representations for the data to see which are most effective.

For the current database consisting of 785 CIFs, on a laptop (HP Elitebook 850 G5, Intel Core i5-7300 CPU @ 2.6 GHz, 2712 MHz, two cores, four logic processors, 8 GB RAM), it takes the program ∼30 s to complete a query. This is acceptable, but will not scale well with larger data sets.

We currently use a brute force approach for finding similarity which will not scale well, requiring more sophisticated and faster database browsing approaches to be found. Prior information from the user can help; for example, a list of chemical elements that the user knows should be present in the sample would cut down the search space. Another possibility would be to adopt the approach of the so-called ‘Hanawalt File’ (Hanawalt *et al.*, 1986 ▸), also used in the early days of the powder diffraction file (PDF) (Gates-Rector & Blanton, 2019 ▸), where only the three most intense reflections of the diffraction pattern were taken into account rather than the full pattern. At the time, this approach made great sense; however the computational power available today makes full pattern comparison possible. As the size of the database increases, we will explore increasing the efficiency of the search, for example, through the use of graph-based search algorithms that can pre-store the similarity between every entry in the database (Johnson *et al.*, 2021 ▸). Algorithms for finding nearest-neighbour connections may then be explored to rapidly find the best solutions without having to traverse the entire graph.

## 5. Conclusions

As a first step towards a more machine-readable literature that will ease literature search and make science more readily available, we have demonstrated a prototype application, *pyDataRecognition*. The program takes a measured powder pattern, together with other relevant metadata, as input and returns information on literature papers that may be relevant to the powder pattern uploaded by the user.

This represents the initial steps towards a more machine-readable literature. However, it has already revealed a number of challenges that need to be overcome moving forward. The CIF format is well defined but is not strictly adhered to or validated, at least when it comes to experimental data in powder CIFs. This results in non-usable information in the CIF file database such as non-numeric values where numeric values are expected. Tools are needed to facilitate the deposition of properly validated data-containing CIF entries in the IUCr database. This work is in progress. Regardless, the simple use case of finding relevant papers given a diffraction pattern already gives a glimpse of many other more advanced capabilities that are possible by going down this route of a machine-readable literature.

## Figures and Tables

**Table 1:** Ranks, scores, DOIs and references for the top-five *pyDataRecognition* database entries shown in Fig. 1 ▸ The ‘user data’ are identical to the rank-1 entry in the table.

Rank	Score	DOI	Reference
1	1.0000	https://doi.org/10.1107/S2052520616015675	Stähli *et al.* (2016 ▸)
2	0.7379	https://doi.org/10.1107/S1600536810014327	Zatovsky *et al.* (2010 ▸)
3	0.4631	https://doi.org/10.1107/S1600536813007848	Strutynska *et al.* (2013 ▸)
4	0.4552	https://doi.org/10.1107/S2052520618004092	Bell & Henderson (2018 ▸)
5	0.4261	https://doi.org/10.1107/S2052520614001140	Zvirgzdins *et al.* (2014 ▸)

**Table 2:** Ranks, scores, DOIs and references for the top-five *pyDataRecognition* database entries shown in Fig. 3 ▸

Rank	Score	DOI	Reference
1	0.5723	https://doi.org/10.1107/S0021889813013253	Iturbe-Zabalo *et al.* (2013 ▸)
2	0.3906	https://doi.org/10.1107/S0108768198017984	Sciau *et al.* (1999 ▸)
3	0.3390	https://doi.org/10.1107/S1600576715000941	Orayech *et al.* (2015 ▸)
4	0.2881	https://doi.org/10.1107/S0108768111039759	Kasunič *et al.* (2011 ▸)
5	0.2485	https://doi.org/10.1107/S0108768109011057	Zhang *et al.* (2009 ▸)

**Table 3:** Ranks, scores, DOIs and references for the top-five *pyDataRecognition* database entries shown in Fig. 4 ▸

Rank	Score	DOI	Reference
1	0.8808	https://doi.org/10.1107/S1600576715000941	Orayech *et al.* (2015 ▸)
2	0.7785	https://doi.org/10.1107/S0021889813013253	Iturbe-Zabalo *et al.* (2013 ▸)
3	0.6855	https://doi.org/10.1107/S0108768109011057	Zhang *et al.* (2009 ▸)
4	0.2859	https://doi.org/10.1107/S0108768112017478	Bereciartua *et al.* (2012 ▸)
5	0.2532	https://doi.org/10.1107/S0108768103019013	Palacios *et al.* (2003 ▸)
